# Role of type 2A phosphatase regulatory subunit B56α in regulating cardiac responses to β-adrenergic stimulation *in vivo*

**DOI:** 10.1093/cvr/cvy230

**Published:** 2018-09-10

**Authors:** Sarah-Lena Puhl, Kate L Weeks, Alican Güran, Antonella Ranieri, Peter Boknik, Uwe Kirchhefer, Frank U Müller, Metin Avkiran

**Affiliations:** 1 School of Cardiovascular Medicine and Sciences, King’s College London British Heart Foundation Centre of Research Excellence, St Thomas’ Hospital, Westminster Bridge Road, London, UK; 2 Institute for Cardiovascular Prevention, Ludwig-Maximilians-University, Pettenkoferstrasse 9b, D-80336 Munich, Germany; 3 Baker Heart and Diabetes Institute, 75 Commercial Road, Melbourne, VIC, Australia; 4 Institut für Pharmakologie und Toxikologie, Universitätsklinikum Münster, Domagkstrasse 12, D-48149 Münster, Germany

**Keywords:** Protein phosphatases, Beta-adrenergic receptors, PP2A, Contractile function, Cardiac hypertrophy

## Abstract

**Aims:**

B56α is a protein phosphatase 2A (PP2A) regulatory subunit that is highly expressed in the heart. We previously reported that cardiomyocyte B56α localizes to myofilaments under resting conditions and translocates to the cytosol in response to acute β-adrenergic receptor (β-AR) stimulation. Given the importance of reversible protein phosphorylation in modulating cardiac function during sympathetic stimulation, we hypothesized that loss of B56α in mice with targeted disruption of the gene encoding B56α (*Ppp2r5a*) would impact on cardiac responses to β-AR stimulation *in vivo*.

**Methods and results:**

Cardiac phenotype of mice heterozygous (HET) or homozygous (HOM) for the disrupted *Ppp2r5a* allele and wild type (WT) littermates was characterized under basal conditions and following acute β-AR stimulation with dobutamine (DOB; 0.75 mg/kg i.p.) or sustained β-AR stimulation by 2-week infusion of isoproterenol (ISO; 30 mg/kg/day s.c.). Left ventricular (LV) wall thicknesses, chamber dimensions and function were assessed by echocardiography, and heart tissue collected for gravimetric, histological, and biochemical analyses. Western blot analysis revealed partial and complete loss of B56α protein in hearts from HET and HOM mice, respectively, and no changes in the expression of other PP2A regulatory, catalytic or scaffolding subunits. PP2A catalytic activity was reduced in hearts of both HET and HOM mice. There were no differences in the basal cardiac phenotype between genotypes. Acute DOB stimulation induced the expected inotropic response in WT and HET mice, which was attenuated in HOM mice. In contrast, DOB-induced increases in heart rate were unaffected by B56α deficiency. In WT mice, ISO infusion increased LV wall thicknesses, cardiomyocyte area and ventricular mass, without LV dilation, systolic dysfunction, collagen deposition or foetal gene expression. The hypertrophic response to ISO was blunted in mice deficient for B56α.

**Conclusion:**

These findings identify B56α as a potential regulator of cardiac structure and function during β-AR stimulation.

## 1. Introduction

Phosphorylation is a post-translational modification that plays a fundamental role in modulating cardiac structural and functional responses to neurohormonal stimuli. Activation of cardiac β-adrenergic receptors (β-ARs) leads to the dynamic phosphorylation and dephosphorylation of myofilament proteins [e.g. cardiac troponin I (cTnI), cardiac myosin binding protein C (cMyBP-C)^1–3^] and calcium handling proteins [e.g. ryanodine receptor 2 (RyR2), phospholamban (PLB), L-type Ca^2+^ channel^4^], as well as transcriptional regulators (e.g. transcription factors,^5^ histone deacetylases^6^^,^^7^). Much research effort to date has focussed on identifying the kinase signalling pathways that facilitate the phosphorylation of functionally important cardiac proteins downstream of physiologically relevant stimuli (see for review, Solaro^8^). However, there is increasing recognition of the importance of dephosphorylation by protein phosphatases in regulating cardiac homeostasis and contractility of the healthy and diseased heart.^9^ Indeed, protein phosphatase expression and activity is dysregulated in the failing heart,^9^ and abnormal phosphorylation of several cardiac proteins that are phospho-regulated in response to β-AR stimulation has been causally associated with various forms of cardiomyopathy.^10^^,^^11^

The type 1 and type 2A protein phosphatases (PP1 and PP2A, respectively) provide the majority of serine/threonine phosphatase activity in the myocardium, and play critical roles in the regulation of cardiac function.^9^^,^^12^^,^^13^ PP2A is a heterotrimeric enzyme consisting of a catalytic C subunit (PP2A_C_, encoded by *PPP2CA* and *PPP2CB*), a scaffolding A subunit (PP2A_A_, encoded by *PPP2R1A* and *PPP2R1B*) and a regulatory B subunit (encoded by at least 15 different genes, including members of the *PPP2R2*, *PPP2R3*, *PPP2R5*, and *STRN* families, with multiple splice variants). The diversity of PP2A cellular functions derives from the fact that numerous biochemically distinct complexes may be assembled from different combinations of A, B, and C subunits, with the regulatory B subunits providing the essential determinants for subcellular targeting, substrate specificity and fine-tuning of phosphatase activity.^14^^,^^15^

B56α is a member of the B56 or B’ family of PP2A regulatory subunits and is most abundant in the heart and skeletal muscle.^16^ We identified B56α as a potential regulator of cardiac β-adrenergic signalling from a proteomic screen in which ventricular myocytes from adult rat hearts were treated with isoproterenol (ISO) to activate β-AR signalling, and then ‘skinned’ to disrupt sarcolemmal membranes and deplete cytosolic proteins.^17^ B56α was found to associate with the Z disc and M-band of sarcomeres in resting cardiomyocytes, and to translocate from the myofilaments to the cytosol in response to acute β-AR stimulation.^17^ We have recently reconfirmed B56α translocation in response to β-AR stimulation in adult rat ventricular myocytes and found that another B56 isoform, B56δ, displays increased phosphorylation (at a unique Ser573 residue) but no translocation following β-AR stimulation in that cell type.^18^ In other pertinent studies, transgenic overexpression of B56α in the murine heart was associated with reduced phosphorylation of myofilament proteins, including cTnI, cMyBP-C, cardiac troponin T, and myosin light chain 2,^19^ and B56α expression in left ventricular (LV) tissue was found to be dramatically reduced in failing human myocardium.^20^ Together, these findings suggest that changes in the abundance or subcellular distribution of B56α may be of functional significance in physiological and pathophysiological settings.

Here, we investigate how loss of B56α impacts on cardiac responses to β-AR activation *in vivo*, using a genetically modified mouse model with targeted disruption of the gene encoding B56α (*Ppp2r5a*). We report that loss of B56α attenuates the inotropic response to acute β-AR stimulation and blunts LV hypertrophy in response to sustained β-AR stimulation. Our findings reveal a potential new role for B56α in the regulation of cardiac structural and functional responses to β-AR activation *in vivo*.

## 2. Methods

### 2.1 Experimental animals

Mice with targeted disruption of *Ppp2r5a* via gene-trap insertion of a BGEO cassette in intron 1 were generated at Texas A&M Institute for Genomic Medicine (TX, USA) and maintained on a C57BL/6 background at St Thomas’ Hospital (London, UK). Mice heterozygous (HET) or homozygous (HOM) for the mutant allele and wild type (WT) littermates were genotyped by polymerase chain reaction (PCR) using forward primers (5′-CTTGCAAAATGGCGTTACTTAAGC-3′,5′-CATCTGGTTAGTCTTGAGATGC-3′) and a reverse primer (5′-TATCAAGGGCACTGCCAAAG-3′) to amplify 370 and 290 bp regions of the WT and mutant alleles, respectively. Male and female mice from all three genotypes and different ages (2 months, 6 months) were included in this study. No signs of infertility, premature mortality or developmental defects were observed. Animal experiments were conducted in accordance with the Home Office Guidance on the Operation of Animals (Scientific Procedures) Act 1986, published by Her Majesty’s Stationary Office, London.

### 2.2 Dobutamine administration

Dobutamine-stress-echocardiography was performed on male WT, HET, and HOM mice as described previously.^21^ In brief, mice were randomly assigned to receive a single bolus intraperitoneal injection of DOB (0.75 mg/kg) or an identical volume of vehicle (0.9% NaCl). Echocardiographic and electrocardiogram (ECG) recordings obtained 10 min after DOB/vehicle administration were analysed.

### 2.3 Isoproterenol infusion

WT and HOM mice received a continuous infusion of ISO (30 mg/kg/day) or vehicle (0.9% NaCl) for 14 days via subcutaneously-implanted osmotic minipumps (ALZET model 1002; supplied by Charles River UK Ltd, Kent, UK), as described previously.^21^ Echocardiographic recordings were obtained prior to minipump implantation and at the end of the infusion period, and hearts were collected for gravimetric, histological and biochemical analyses. ISO (DL-Isoproterenol hydrochloride) was from Sigma (MO, USA).

### 2.4 Echocardiography

Echocardiography was performed on anaesthetized mice (1.5–2% isofluorane) using the VisualSonicsVevo^®^ 770 imaging system (Scanhead: RMV707B, 15–45 MHz, cardiac mouse), as described previously.^21^ End-systolic and end-diastolic measurements of interventricular septum (IVS) thickness, LV internal dimension (LVID) and LV posterior wall (LVPW) thickness were obtained from parasternal long-axis projections, and LV anterior wall (LVAW) thickness was obtained from short-axis projections. Measurements of LV dimensions were obtained from three beats and averaged. LV fractional shortening (LVFS) [(LVID; d - LVID; s)/LVID; d*100%] and LV ejection fraction (LVEF) [(LVvol; d - LVvol; s)/LVvol; d*100%] were calculated from M-mode measurements. ECG and respiration gating were used to suppress movement artefacts.

### 2.5 Tissue sampling and processing

Mice were anaesthetized with 4% isoflurane and killed by cervical dislocation. Tissue collection and processing was performed as described previously.^22^ In brief, hearts were removed, washed in cold PBS and patted dry with sterile gauze prior to weighing. Mid-ventricular slices were fixed in 4% paraformaldehyde and stored at 4°C for histological analyses. The remaining ventricular tissue was snap-frozen in liquid N_2_ and stored at −80°C for biochemical analyses. The lungs, liver, spleen and left kidney were weighed following removal (wet weight) and after dehydration at 50°C overnight (dry weight) for assessment of tissue congestion.

### 2.6 Protein phosphatase assay

Protein phosphatase activity was determined with [^32^P]-phosphorylase *a* as substrate and 3 nmol/L okadaic acid was used to differentiate between PP1 and PP2A activity, as described previously.^19^^,^^20^

### 2.7 Histology

LV collagen content (fibrosis) and myocyte cross-sectional area were quantified from transverse sections of the left ventricle, as described previously.^23^ In brief, mid-ventricular tissue slices fixed in 4% paraformaldehyde and embedded in paraffin were sectioned (5 μm) and stained with picrosirius red or haematoxylin and eosin (H&E). Quantification of LV collagen content (from picrosirius red-stained sections) and myocyte cross-sectional area (from H&E-stained sections) was performed using Image Pro Plus 7.0 and Image J software, respectively.

### 2.8 SDS-PAGE and immunoblotting

Pulverized LV tissue was homogenized on ice in PBS supplemented with EDTA-free protease inhibitor (Roche 11873580001) and phosphatase inhibitor cocktail 3 (Sigma P0044) and lysed in sample buffer at 95°C for 10 min. Proteins were resolved on Tris-glycine SDS-PAGE gels and transferred to polyvinylidene difluoride membranes. Phosphate affinity (PhosTag) SDS-PAGE was performed on Tris-glycine gels containing acrylamide-pendant PhosTag and MnCl_2_, as previously described.^18^^,^^24^^,^^25^ Immunoblot analyses were performed using the following antibodies: PP2A_Cα_ (1:1000, Proteintech 13482-1-AP), PP2A_Cα/β_ (1:2000, Cell Signaling 2038), PP2A_A_ (1:1000, Santa Cruz sc-74580) B56α (1:500, BD Transduction 610615), B56γ (1:500, Abcam ab94633), B56δ (1:1000, Bethyl A301-100A), B55α (1:500, Santa Cruz sc-81606), α4 (1:2000, Bethyl A300-471A), phospho-PKA substrate RRXS/T (1:2000, Cell Signaling 9624), cTnI (1:2000, Cell Signaling 2002), pS16 PLB (1:4000, Badrilla A010-12AP), PLB (1:1000, Cell Signaling 8495), pS282 cMyBP-C (Enzo Life Sciences ALX-215-057-R050), RyR2 (1:500, Thermo Fisher Scientific MA3-925), α-actinin (1:10 000, Abcam ab50599), GAPDH (1:1000, Cell Signaling 2118). The cMyBP-C and pS302 cMyBP-C antibodies were kind gifts from Gautel *et al.*^26^ and Robbins *et al.*,^27^ and were used at 1:30 000 and 1:5000, respectively. The pS2808 and pS2814 RyR2 antibodies were kind gifts from Wehrens *et al.*^28^ and were used at 1:1000. The following HRP-linked secondary antibodies were used: anti-rabbit IgG (1:2500, Cell Signaling 7074), anti-rat IgG (1:2500, Cell Signaling 7077), anti-mouse IgG (1:2500, Dako P0447). Protein bands were visualized on chemiluminescence film, following the use of ECL Western Blotting Detection Reagents (GE Healthcare). Signals were quantified using Image J software (NIH; phospho-PKA substrate blots) or on a calibrated GS-800 densitometer (Bio-Rad; all other blots). Cardiac PP2A subunit expression was normalized to α-actinin. Ventricular tissue treated with λ-phosphatase^29^ was included on PhosTag SDS-PAGE gels to confirm the specificity of the bands corresponding to mono- and bis-phosphorylated cTnI.

### 2.9 Quantitative reverse transcription PCR

LV tissue was homogenized, lysed and RNA isolated via phenol extraction. cDNA obtained from 1 µg RNA was diluted five-fold for quantitative SYBR Green PCR analysis and 10-fold for TaqMan PCR analysis. Samples were run in triplicate, and mRNA expression normalized to *Gapdh*. Relative gene expression was calculated using the comparative ΔΔCT method. Results are presented as fold-change vs WT or WT Vehicle as appropriate. TaqMan probe/primer sets were from Applied Biosystems: *Nppb*: Mm01255770_g1; *Gapdh*: Mm99999915_g1. The following primers were used for SYBR Green PCR analysis: *Myh6*: for AACTACCACATCTTCTACC, rev TAGTCGTATGGGTTGTTG; *Myh7*: for GCTGTTATTGCCGCCATTG, rev GTTGTCATTCCGAACTGTCTTG; *Nppa*: for GGCTCCTTCTCCATCACC, rev CGGCATCTTCTCCTCCAG; *Col1a1:* for GCTCCTCTTAGGGGCCACT, rev CCACGTCTCACCATTGGGG; *Col3a1*: for CTGTAACATGGAAACTGGGGAAA, rev CCATAGCTGAACTGAAAACCACC; *Gapdh*: for AGGTCGGTGTGAACGGATTTG, rev GGGGTCGTTGATGGCAACA.

### 2.10 Statistical analyses

Quantitative data are presented as mean ± standard error (SE). Differences between groups were determined by unpaired *t*-test, or by one- or two-way ANOVA with Tukey’s *post hoc* test, as appropriate. A *P-*value less than 0.05 was considered significant. All statistical analyses were performed with Microsoft Excel 2007 and Graph Pad Prism 6.0 software.

## 3. Results

**Figure 1:**
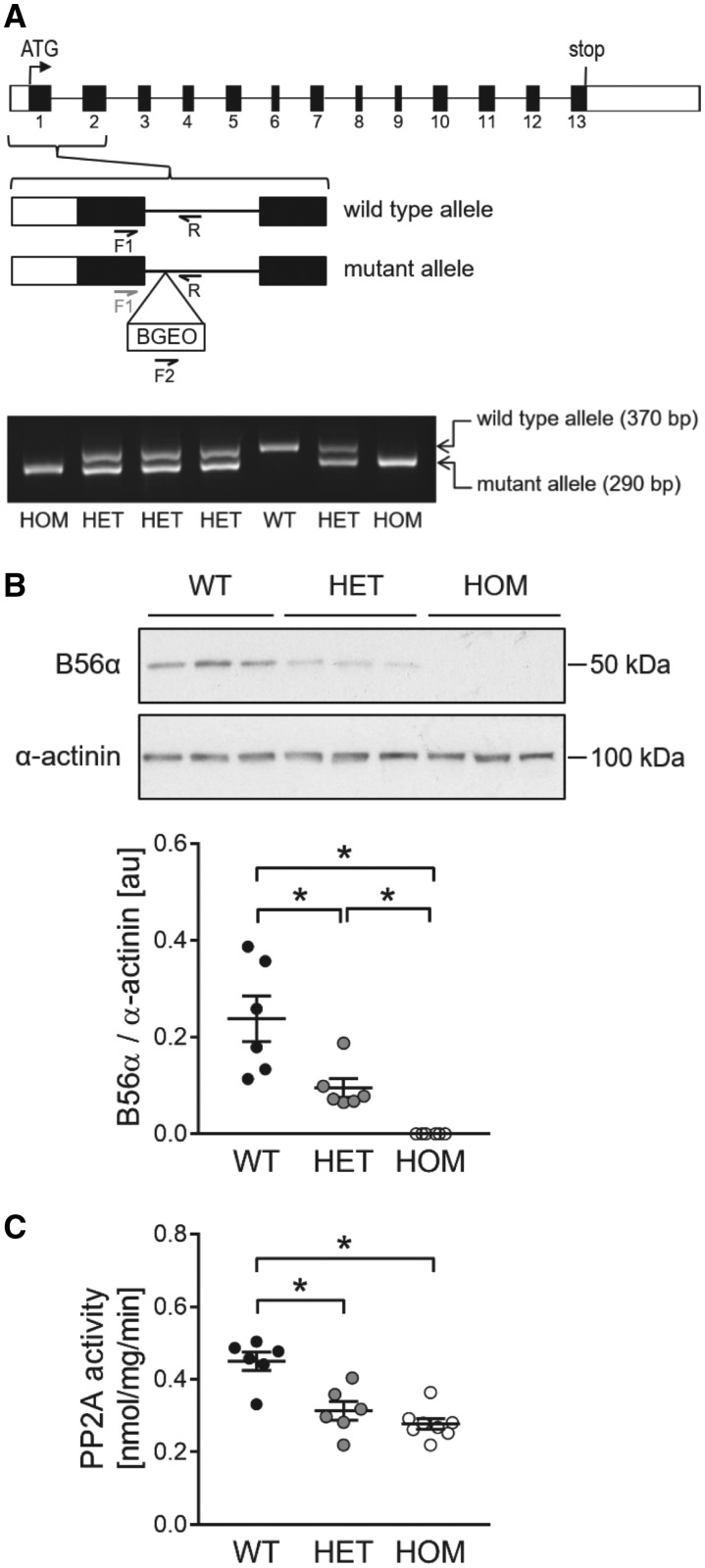
Generation and characterization of mice deficient for B56α. (*A*) Schematic of the gene encoding B56α (*Ppp2r5a*; exons in black) showing the insertion site of the gene trap cassette (BGEO) and locations of forward (F1, F2) and reverse (R) primers used to amplify regions of the WT and mutant alleles by PCR from genomic DNA of WT, HET, and HOM mice. A DNA gel illustrating the outcome of a typical genotyping run is also shown. (*B*) Protein levels of B56α in ventricular tissue from WT, HET, and HOM mice, assessed by immunoblotting (*n *=* *6 per group). (*C*) PP2A catalytic activity in ventricular tissue from WT, HET, and HOM mice, assessed by a quantitative assay that uses ^32^P-labelled phosphorylase *a* as substrate (*n* = 6–8 per group). Lines show mean ± SE; *P <* *0.05 (one-way ANOVA with Tukey’s *post hoc* test).

To explore the *in vivo* roles of B56α in regulating cardiac structure and function, we utilized a mouse line with targeted disruption of *Ppp2r5a*, the gene encoding B56α. This was achieved through a gene trap approach, with the trapping cassette inserted in intron 1 (*Figure 1A*). As illustrated in *Figure 1B*, relative to WT mice, targeted disruption of *Ppp2r5a* gave rise to a partial loss of cardiac B56α protein in mice heterozygous for the mutant allele (HET) and complete loss of cardiac B56α protein in mice HOM for the mutant allele (HOM). Interestingly, relative to WT mice, cardiac PP2A activity was found to be significantly reduced in both HET and HOM mice, with a somewhat greater reduction in the latter (*Figure 1C*).

**Figure 2:**
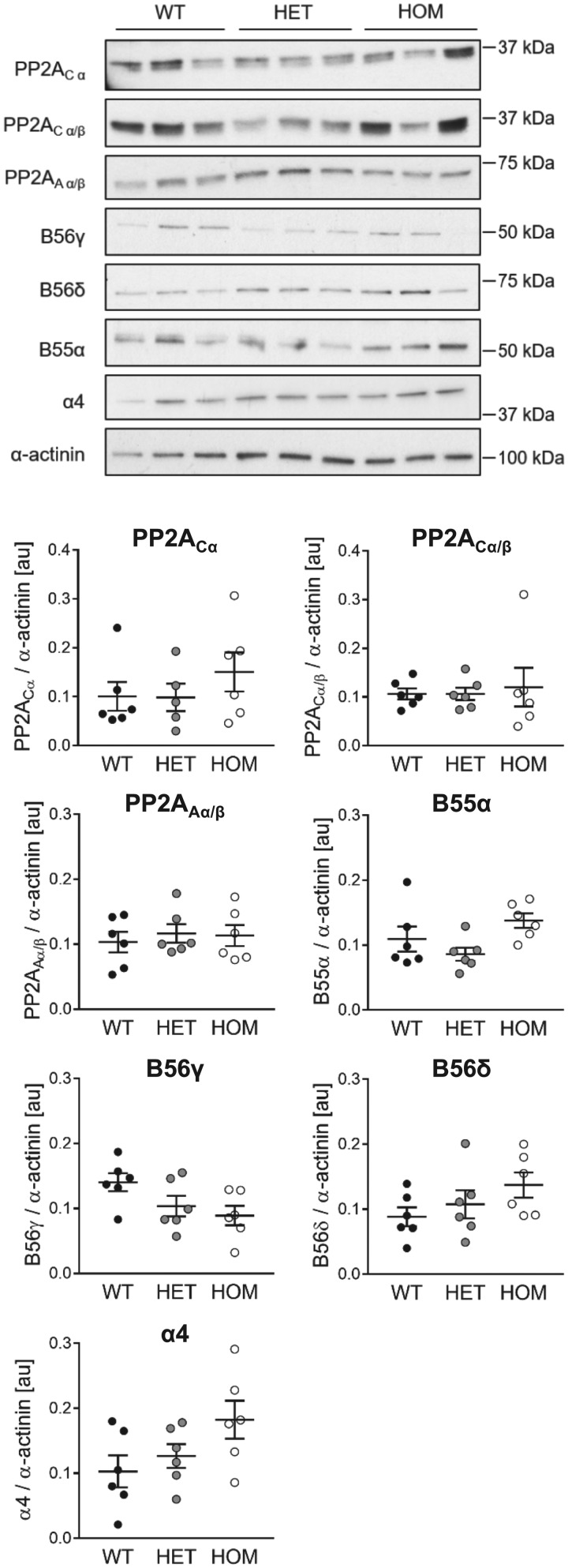
Cardiac expression of PP2A catalytic, scaffolding and regulatory subunits. Protein levels of PP2A_C_ α isoform, PP2A_C_ α and β isoforms, PP2A_A_ α and β isoforms, B55α, B56γ, B56δ, and α4 in ventricular tissue from WT, HET, and HOM mice, assessed by immunoblotting. Lines show mean ± SE (*n *=* *5–6 per group). There were no significant differences between groups (one-way ANOVA).

To explore if the loss of B56α protein was associated with altered expression of other components of the heterotrimeric PP2A holoenzyme, we explored the protein levels of PP2A catalytic (PP2A_C_) and scaffolding (PP2A_A_) subunits, and three other PP2A regulatory B subunit isoforms (B56δ, B56γ, and B55α), in hearts from WT, HET, and HOM mice (*Figure 2*). In the same samples, the expression of α4, an evolutionarily conserved PP2A_C_-binding protein,^30^ was also determined (*Figure 2*). Cardiac expression of PP2A_C_ (determined using antibodies that recognize only the α isoform or both the α and β isoforms) and PP2A_A_ (determined using an antibody that recognizes both the α and β isoforms) was comparable in WT, HET, and HOM mice. There was also no significant difference between genotypes in the cardiac expression of B56γ, B56δ, or B55α, although a trend towards reciprocal changes in the expression of B56γ (decreased) and B56δ (increased) with increasing number of mutant *Ppp2r5a* alleles was observed (*Figure 2*). Interestingly, there was also a strong trend towards increased abundance of α4 occurring in parallel with the decreased abundance of B56α in HET and HOM mice (*Figure 2*).

**Figure 3:**
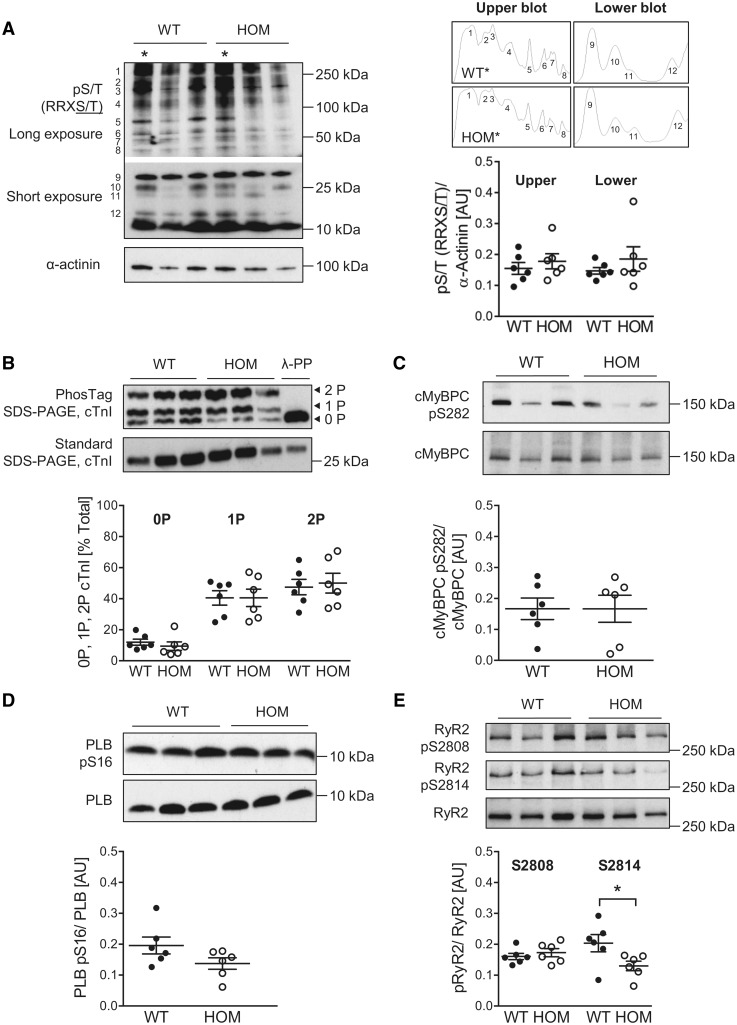
Cardiac protein phosphorylation in WT and B56α-deficient mice. (*A*) Phosphorylation of Ser/Thr residues within the optimal protein kinase A substrate motif, assessed by immunoblotting with a pS/T (RRXS/T) antibody. Discrete bands on the immunoblot are numbered. Plots generated using a gel analysis tool (Image J software, NIH) show the peaks corresponding to each band in the WT and HOM samples marked with an asterisk (*). The overall phosphorylation signal for large (>37 kDa, upper blot) and small (<37 kDa, lower blot) proteins was quantified by measuring the area under the curve for each plot and normalizing to α-actinin. Lines show mean ± SE (*n *=* *6 per group). There were no significant differences between genotypes (unpaired *t*-tests). (*B*) Abundance of non-phosphorylated (0P), mono-phosphorylated (1P) and bis-phosphorylated (2P) cTnI, as assessed by phosphate affinity (PhosTag) SDS-PAGE and immunoblotting. 0P, 1P, and 2P phospho-moieties are expressed as a percentage of total cTnI, which is the sum of the signals in each lane. The specificity of the bands was confirmed by incubating with λ-phosphatase (λ-PP) to dephosphorylate cTnI. Lines show mean ± SE (*n *=* *6 per group); *P <* *0.05 (two-way ANOVA with Tukey’s *post hoc* test). (*C*) Phosphorylation of cardiac myosin binding protein C (cMyBP-C) at Ser282, as assessed by immunoblotting. Lines show mean ± SE (*n *=* *6 per group). There was no significant difference between the genotypes (unpaired *t*-test). (*D*) Phosphorylation of phospholamban (PLB) at Ser16, as assessed by immunoblotting. Lines show mean ± SE (*n *=* *6 per group). There was no significant difference between genotypes (unpaired *t-*test). (*E*) Phosphorylation of RyR2 at Ser2808 and Ser2814, as assessed by immunoblotting. Lines show mean ± SE (*n *=* *6 per group); *P <* *0.05 (unpaired *t*-test).

We also determined the phosphorylation profile of a number of phosphoproteins in hearts from WT and HOM mice. The use of a phosphospecific antibody that detects phosphorylated Ser/Thr residues within the optimal protein kinase A substrate motif (RRXS/T) allowed the detection of 12 discrete bands, which are likely to represent abundantly expressed phosphoproteins that possess one or more such residue(s) (*Figure 3A*). However, there appeared to be no striking difference between hearts from WT and HOM mice in the intensity of the signals from individual bands or the overall phosphorylation signal (*Figure 3A*). We also explored the site-specific phosphorylation of two functionally important myofilament phosphoproteins, cTnI and cMyBP-C, and two phosphoproteins that regulate Ca^2+^ uptake into and release from the sarcoplasmic reticulum, PLB and RyR2. This analysis revealed no significant difference between hearts from WT and HOM mice in the phosphorylation status of the majority of the pertinent proteins and residues (*Figure 3B–D*). A notable exception was Ser2814 in RyR2, which exhibited significantly lower phosphorylation in hearts of HOM mice (*Figure 3E*).

Complementary analyses of protein phosphorylation were performed in ventricular myocytes isolated from the hearts of adult WT and HOM mice and subjected to stimulation with vehicle or ISO *in vitro*. Consistent with our observations in mouse hearts, loss of B56α had no significant effect on the phosphorylation of cTnI, cMyBP-C or PLB in vehicle-treated cells (see Supplementary material online, *Figure S1A–C*). The phosphorylation of RyR2 tended to be lower in myocytes from HOM mice and was not altered by ISO stimulation in either genotype (see Supplementary material online, *Figure S1D*). ISO stimulation increased the abundance of mono- and bis-phosphorylated cTnI while reducing the abundance of non-phosphorylated cTnI, and increased the phosphorylation of cMyBP-C at Ser282 and Ser302 and PLB at Ser16, in myocytes from both WT and HOM mice (see Supplementary material online, *Figure S1A–C*).

**Figure 4:**
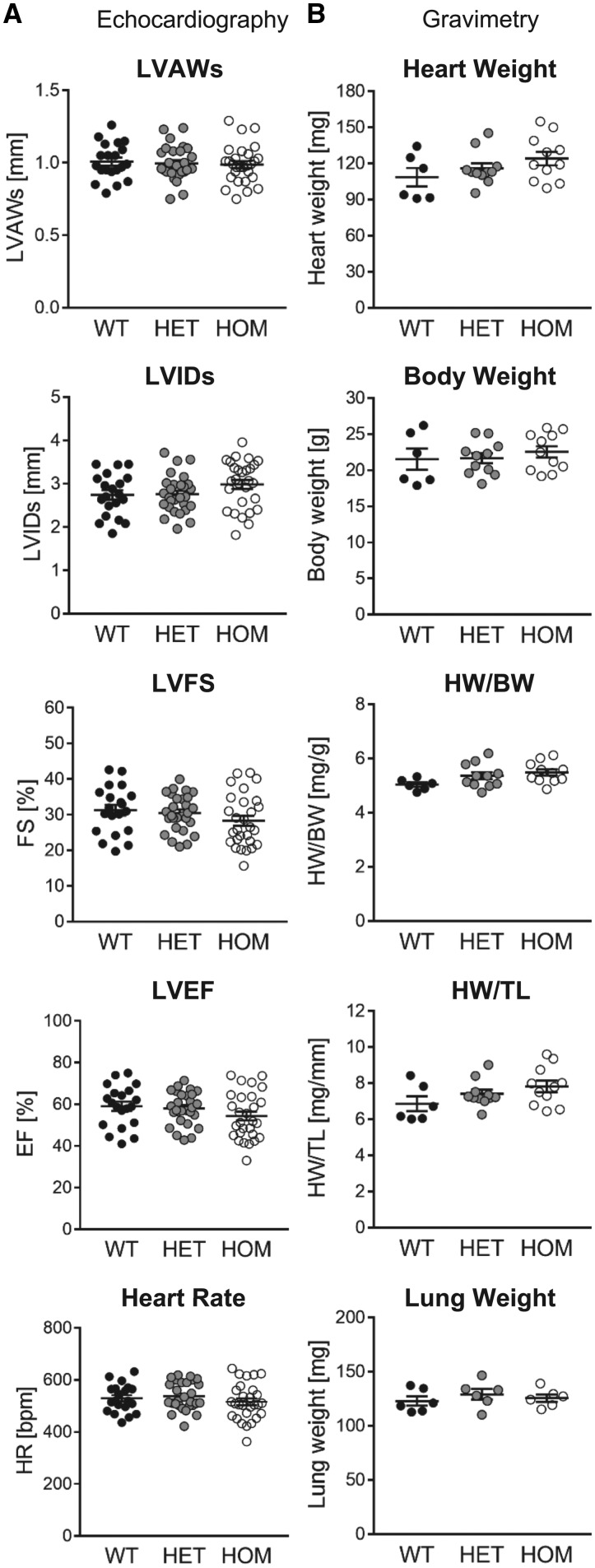
Echocardiographic and gravimetric analysis of basal cardiac phenotype. (*A*) Echocardiographic assessment of LV anterior wall thickness at end-systole (LVAWs), LV internal dimension at end-systole (LVIDs), LV fractional shortening (LVFS), LV ejection fraction (LVEF), and heart rate (HR) in male WT, HET, and HOM mice at 8 weeks of age. Lines are mean ± SE (*n *=* *20–28 per group). There were no significant differences between groups (one-way ANOVA). (*B*) HW, body weight (BW), HW normalized to BW and TL, and LW of male WT, HET, and HOM mice at 8 weeks of age. Lines show mean ± SE (*n *=* *6–11 per group for all parameters except LW, for which *n *=* *6 per group). There were no significant differences between groups (one-way ANOVA).

Basal cardiac structure and function *in vivo* were assessed by echocardiographic analysis in anaesthetized mice at 2 months of age. This revealed no significant difference between genotypes in LV wall thicknesses, LV cavity size, LV contractile function or heart rate, in either sex (*Figure 4A* and Supplementary material online, *Tables S1 and S2*). Gravimetric analyses also did not reveal any significant variation between genotypes in body or organ weight, in either sex (*Figure 4B* and Supplementary material online, *Tables S3 and S4*). At 6 months of age, male HOM mice displayed a small, but significant, decrease in heart weight (HW) normalized to tibia length (TL; ∼12% decrease vs. WT; Supplementary material online, *Table S5*). This was accompanied by a comparable decrease in lung weight (LW) normalized to TL (see Supplementary material online, *Table S5*). By echocardiography, IVS thickness was also reduced in the HOM mice (see Supplementary material online, *Table S6*), consistent with the decrease in HW/TL ratio. These differences were not apparent in 6-month old female mice (see Supplementary material online, *Tables S7 and S8*).

**Figure 5:**
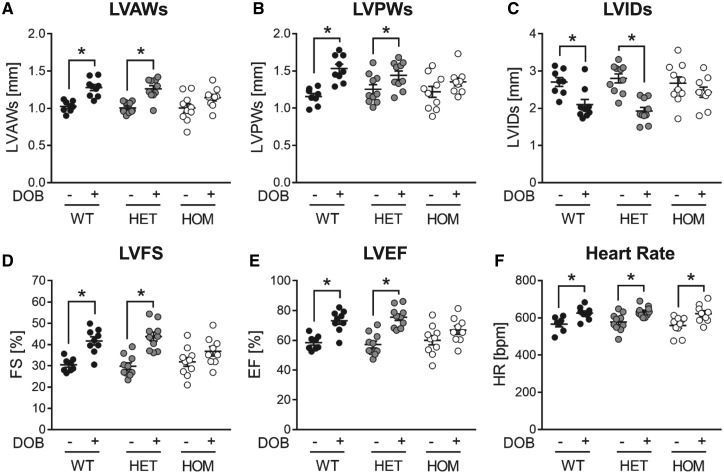
Echocardiographic analysis of cardiac responses to acute β-adrenergic stimulation. Echocardiographic assessment of (*A*) LV anterior wall thickness at end-systole (LVAWs), (*B*) LV posterior wall thickness at end-systole (LVPWs), (*C*) LV internal dimension at end-systole (LVIDs), (*D*) LV fractional shortening (LVFS), (*E*) LV ejection fraction (LVEF), and (*F*) heart rate (HR) in male WT, HET, and HOM mice following intraperitoneal injection of DOB (0.75 mg/kg) or vehicle. Lines show mean ± SE (*n *=* *8–10 per group); *P <* *0.05 (two-way ANOVA with Tukey’s *post hoc* test).

Based on our previous work indicating that, in isolated adult rat ventricular cardiomyocytes, β-AR stimulation induces B56α translocation,^17^ we explored the impact of the loss of B56α protein on cardiac responses to acute β-AR stimulation *in vivo*, again by echocardiographic analysis. This revealed that acute β-AR stimulation with dobutamine induced the predicted positive inotropic and positive chronotropic responses in WT and HET mice (*Figure 5* and Supplementary material online,* Table S9*). Interestingly, however, the positive inotropic response was attenuated while the positive chronotropic response was retained in HOM mice (*Figure 5* and Supplementary material online, *Table S9*).

**Figure 6:**
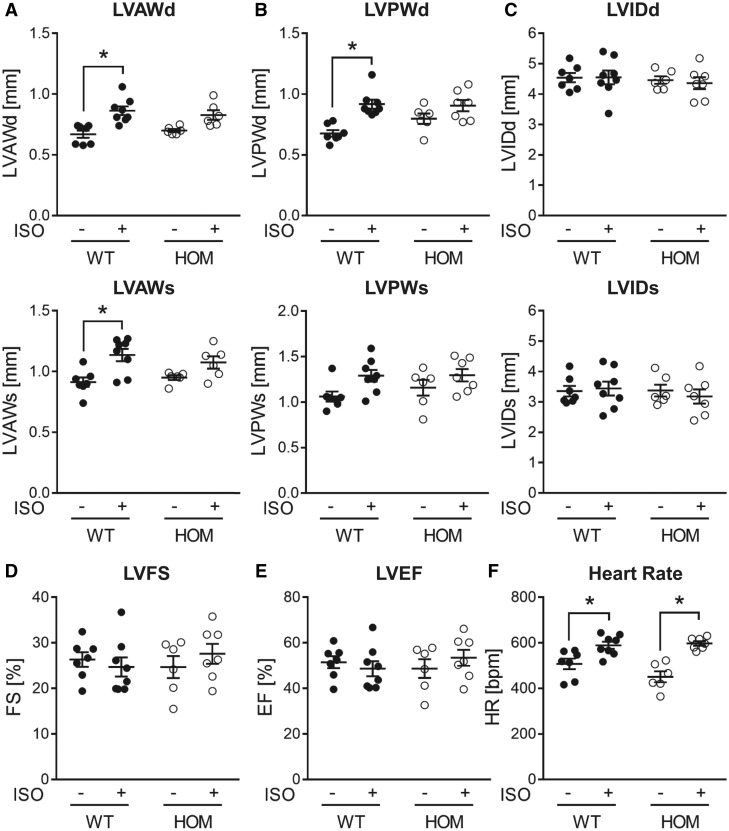
Echocardiographic analysis of cardiac responses to sustained β-adrenergic stimulation. Echocardiographic assessment of (*A*) LV anterior wall thickness (LVAW), (*B*) LV posterior wall thickness (LVPW), (*C*) LV internal dimension (LVID) at end-diastole (d) and end-systole (s), (*D*) LV fractional shortening (LVFS), (*E*) LV ejection fraction (LVEF), and (*F*) heart rate in male WT and HOM mice following subcutaneous infusion of ISO (30 mg/kg/day) or vehicle for 14 days. Lines show mean ± SE (*n *=* *6–8 per group); *P <* *0.05 (two-way ANOVA with Tukey’s *post hoc* test).

The differential cardiac inotropic response to acute β-AR stimulation in HOM mice prompted us to also investigate whether cardiac structural and functional responses to sustained β-AR stimulation are altered in this genotype. To that end, WT and HOM mice received subcutaneous infusion of saline (Control) or isoproterenol (ISO), delivered through implanted osmotic mini-pumps, for a 2-week period. As shown in *Figure 6*, in WT mice, ISO infusion induced significant thickening of the LV anterior and posterior walls at end-diastole, without a significant change in LV chamber size, which is indicative of LV hypertrophy without LV dilation. In HOM mice, the ISO-induced increases in LV wall thickness were much less marked and did not reach statistical significance relative to genotype-matched control mice receiving saline (*Figure 6*), suggesting attenuated LV hypertrophy. Similar to our observations with acute β-AR stimulation using dobutamine (*Figure 5*), there was a significant positive chronotropic response to ISO in both WT and HOM mice (*Figure 6*). There were no significant differences between groups in other echocardiographic parameters (*Figure 6* and Supplementary material online, *Table S10*).

**Figure 7:**
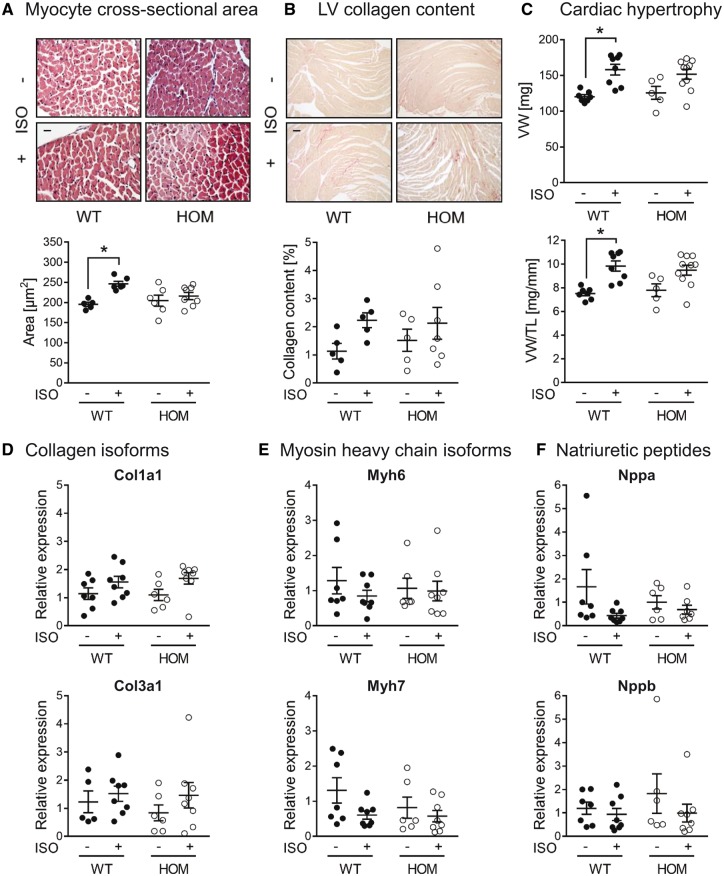
Histological, gravimetric and molecular analysis of cardiac responses to sustained β-adrenergic stimulation. (*A*) Cross-sections of the LV stained with haematoxylin and eosin to allow quantification of myocyte cross-sectional area. Representative images (magnification ×400) and quantitative data from male WT and HOM mice following subcutaneous infusion of ISO (30 mg/kg/day) or vehicle for 14 days. Scale bar: 20 μm. Lines show mean ± SE (>50 myocytes were measured from 5 to 8 hearts per group); *P <* *0.05 (two-way ANOVA with Tukey’s *post hoc* test). (*B*) Cross-sections of the LV stained with picrosirius red to allow quantification of LV collagen content. Representative images (magnification ×100) and quantitative data from male WT and HOM mice following subcutaneous infusion of ISO (30 mg/kg/day) or vehicle for 14 days. Scale bar: 80 μm. Lines show mean ± SE (*n *=* *5–7 per group). There were no significant differences between groups (two-way ANOVA). (*C*) VW and VW normalized to TL of male WT and HOM mice following subcutaneous infusion of ISO (30 mg/kg/day) or vehicle for 14 days. Lines show mean ± SE (*n *=* *5–10 per group); *P <* *0.05 (two-way ANOVA with Tukey’s *post hoc* test). (*D*–*F*) Quantitative reverse transcription PCR analysis of *Col1a1*, *Col3a1*, *Myh6*, *Myh7*, *Nppa*, and *Nppb* mRNA transcript levels in hearts from male WT and HOM mice following subcutaneous infusion of ISO (30 mg/kg/day) or vehicle for 14 days. Lines show mean ± SE (*n *=* *5–8 per group). There were no significant differences between groups (two-way ANOVA).

We also investigated ISO-induced LV remodelling in WT vs. HOM mice by histological and gravimetric analyses. ISO infusion for 2 weeks led to a significant increase in LV myocyte cross-sectional area in WT mice, but not in HOM mice (*Figure 7A*). There was little detectable LV collagen in WT or HOM mice, as measured by picrosirius red staining, in the absence or presence of ISO infusion (*Figure 7B*). Ventricular weight (VW), in absolute terms or when normalized to TL, was significantly increased by ISO in WT mice, but not in HOM mice (*Figure 7C*). Gravimetric analyses of other organs did not reveal major differences between genotypes, except that ISO infusion induced a significant increase in LW in HOM but not WT mice (see Supplementary material online, *Table S11*).

Gene expression analyses revealed that there was no significant difference between groups in the expression of *Col1a1* or *Col3a1*, assessed as molecular markers of the induction of cardiac fibrosis (*Figure 7D*). Similarly, there was no significant difference between groups in molecular markers of the induction of pathological cardiac hypertrophy, such as reciprocal changes in the expression of the myosin heavy chain genes *Myh6* and *Myh7* (*Figure 7E*) or increased expression of the natriuretic peptide genes *Nppa* and *Nppb* (*Figure 7F*).

## 4. Discussion

Our study provides an extensive characterization of the impact of disrupting B56α expression on cardiac phenotype in mice, with focus on contractile and remodelling responses to acute and sustained β-AR stimulation, respectively. Our data indicate that loss of B56α does not influence resting cardiac structure and function, but it attenuates the cardiac inotropic response to acute β-AR stimulation and blunts the cardiac hypertrophy that arises from sustained β-AR stimulation. These observations build on our previous work that revealed translocation of B56α between subcellular compartments upon β-AR stimulation in cardiomyocytes^17^^,^^18^ and suggest that B56α plays an important role in regulating cardiac responses to such stimulation *in vivo*.

The role of B56α in regulating the inotropic response to acute β-AR stimulation was revealed by our observation of attenuated dobutamine-induced increases in LVFS and LVEF in HOM mice relative to their WT littermates (*Figure 5*). Interestingly, heart rate was increased significantly and to a similar extent in both WT and HOM mice in response to dobutamine, indicating that proximal β-AR signalling events, such as activation of adenylate cyclase by stimulatory G proteins, elevation of intracellular cAMP, and activation of cAMP-dependent protein kinase (PKA), are unaffected by the loss of B56α, at least in sinoatrial node pacemaker cells. An attenuated inotropic response to acute β-AR stimulation may arise from altered phosphorylation of myofilament or Ca^2+^-regulatory protein(s) in cardiomyocytes.^4^ Of note, we found no difference between hearts from WT and HOM mice in the phosphorylation status of cTnI, cMyBP-C, or PLB (*Figure 3B–D*), suggesting that these phosphoproteins are not regulated by B56α-PP2A. Interestingly, RyR2 phosphorylation was unaffected at Ser2808 (a PKA target residing in a TRRISQTSQ motif) but was significantly reduced at Ser2814 [a Ca^2+^/calmodulin-dependent kinase II (CaMKII) target residing in a TSQVSIDAA motif] in hearts from HOM mice relative those from WT mice (*Figure 3E*). It is important to note that, although these protein phosphorylation analyses were performed using hearts from mice that did not receive treatment with an exogenous β-AR agonist, euthanasia in mice is associated with marked sympathetic activation, as reflected by the ability of β-AR antagonists to markedly attenuate the phosphorylation of both cTnI and cMyBP-C when administered *in vivo* prior to euthanasia.^31^ Indeed, our observation of marked cTnI phosphorylation in WT hearts, in which approximately 50% of cTnI existed in the bis-phosphorylated (2P) state (*Figure 3B*), attests to significant sympathetic activation prior to heart excision. Taken together, our findings suggest that, while altered cTnI, cMyBP-C, or PLB phosphorylation is unlikely to play a role, reduced RyR2 phosphorylation at Ser2814 may contribute to the attenuated inotropic response to acute β-AR stimulation that we have observed in HOM mice. Nevertheless, this requires confirmation by further investigation, particularly in view of reservations regarding the importance of RyR2 phosphorylation at Ser2808 in mediating the positive inotropic effects of β-AR stimulation,^32–34^ and our observation that in ventricular myocytes isolated from either WT or HOM mice acute β-AR stimulation with ISO did not induce a significant change in RyR2 phosphorylation (see Supplementary material online, *Figure S1*).

With regard to the pertinent molecular signalling mechanism, overexpression of the microRNA miR-1 in adult rat ventricular myocytes has been shown both to reduce B56α expression and to increase RyR2 phosphorylation at Ser2814, leading to the suggestion that RyR2 may be dephosphorylated at this site by B56α-PP2A.^35^ However, our current data and recent evidence from a similar model^36^ show that loss of B56α protein is associated with reduced, rather than increased, RyR2 phosphorylation at S2814 in mouse hearts, suggesting that this site is not a direct target for B56α-PP2A *in vivo*. It is likely, therefore, that any B56α-PP2A-mediated regulation of RyR2 phosphorylation occurs through an indirect mechanism, potentially through a downstream change in PP1 activity.^37^

The blunted hypertrophic response to sustained β-AR stimulation in mice lacking B56α might arise, at least in part, from a diminished increase in cardiac workload, due to the attenuated inotropic response to such stimulation. Nevertheless, during sustained β-AR stimulation other pathways are also activated, including for example CaMKII and its downstream signalling mechanisms that precipitate pathological remodelling.^38^^,^^39^ Whether B56α-PP2A is required for such signalling is currently unknown. It is worth noting, however, that the ISO-induced hypertrophy that we have observed in both WT and HOM mice was mild, with no indication of pathological remodelling as characterized by ventricular dilation (there was no significant increase in LVID at diastole or systole), increased interstitial fibrosis (there was no significant increase in *Col1a1* or *Col3a1* gene expression or LV collagen content), reduced LV function (there was no significant reduction in LVFS or LVEF), and up-regulation of foetal genes (there was no significant change in myosin heavy chain or natriuretic peptide gene expression). The impact of B56α deletion on cardiac phenotype following a more severe or prolonged stress stimulus remains to be determined. In this context, B56α expression has been reported to be significantly reduced in LV myocardium of failing human hearts,^20^ although whether this is a causal or compensatory change is unknown. It is also worth noting that knock-in mice that express a mutated RyR2 protein that cannot be phosphorylated at Ser2814 (Ser2814Ala) appear resistant to pathological cardiac remodelling that is induced by transverse aortic constriction^40^ or a 4-week infusion of ISO.^38^

In our studies, reduced cardiac B56α expression in HET and HOM mice was associated with reduced cardiac PP2A catalytic activity. A parallel reduction in cardiac B56α expression and PP2A catalytic activity has been reported previously in a mouse model of endotoxaemia.^41^ Furthermore, a 2-fold increase in cardiac B56α expression in transgenic mice was found to be associated with a parallel increase in PP2A catalytic activity.^19^ Taken together with our data, these studies suggest that in mouse myocardium B56α abundance is an important determinant of PP2A catalytic activity, most likely in specific subcellular compartments where the B56α-PP2A holoenzyme is localized. Nevertheless, Little *et al.*^36^ have reported *increased* PP2A activity in mouse hearts with reduced B56α expression, using a similar model with targeted *Ppp2r5a* disruption. The reason for these discrepant findings is unclear. However, it is worth noting that while the three studies reporting a reduced PP2A activity (Marshall *et al.*,^41^ Kirchhefer *et al.*^19^ and our present study) used protein or peptide substrates with phosphorylated Ser/Thr residues in their enzymatic assays, the study showing an increased PP2A activity^36^ used *p*-nitrophenyl phosphate, which is a chromogenic substrate targeted by various phosphatases, including protein tyrosine phosphatases and alkaline phosphatases.

The reduction in PP2A catalytic activity that we have observed occurred in the absence of a change in the expression of C subunits, which was determined using antibodies recognizing either the α isoform alone or both the α and β isoforms. This points towards a post-translational mechanism for the observed reduction in cardiac PP2A activity in our mouse model. It is well established that B56α is abundantly expressed in the heart,^16^^,^^42^ such that a partial or complete loss of B56α in HET and HOM hearts, respectively, might ‘release’ a significant amount of C subunit that, unless sequestered by other regulatory proteins, could lead to uncontrolled PP2A activity. An important C subunit interacting protein that is distinct from B type regulatory subunits is α4, which is expressed in mouse myocardium^43^ and has been suggested to act as a binding partner for monomeric PP2A C subunits that prevents their unregulated catalytic activity.^30^^,^^44^ Interestingly, we have found that the graded reductions in B56α expression and PP2A catalytic activity in WT, HET and HOM hearts (*Figure 1*) were accompanied by a strong trend towards a reciprocal increase in α4 abundance (*Figure 2*). It is possible, therefore, that increased sequestration of PP2A C subunits by α4 contributed, at least in part, to the reduced PP2A catalytic activity that we have observed in HET and HOM hearts.

## 5. Conclusion

In conclusion, our study has revealed that B56α modulates the cardiac inotropic response to acute β-AR stimulation and the cardiac hypertrophy response to sustained β-AR stimulation in the *in vivo* setting. Taken together with previous evidence that: (i) B56α translocates between subcellular compartments upon β-AR stimulation in cardiomyocytes^17^^,^^18^; (ii) overexpression^19^ or disruption^36^ of B56α expression in mice has a marked impact on cardiac phenotype; and (iii) there is a marked reduction in B56α expression in failing human hearts,^20^ these findings indicate that B56α-PP2A may be a novel regulator of cardiac structure and function. Delineating the molecular mechanism(s) through which B56α-PP2A regulates cardiac structure and function, particularly in response to β-AR stimulation, requires further investigation, including the identification of putative B56α-PP2A substrates through broad spectrum phosphoproteomic analysis.

## Funding

This work was supported by the British Heart Foundation, through a Project Grant (PG/12/48/29638) and a Research Excellence Award (RE/13/2/30182). K.L.W. was supported by an Overseas Research Fellowship from the National Heart Foundation of Australia (O12M6802).

**Conflict of interest:** none declared.

## Supplementary Material

Supplementary Data
