# Supplementary material for: Role of type 2A phosphatase regulatory subunit B56α in regulating cardiac responses to β-adrenergic stimulation in vivo
Source: Cardiovasc Res. 2018 Sep 10;115(3):519–29. doi: 10.1093/cvr/cvy230 (PMC6383118; doi:10.1093/cvr/cvy230)
Supplement: Supplementary Data [file cvy230_supplementary_data.pdf]

SUPPLEMENTARY INFORMATION

Role of type 2A phosphatase regulatory subunit B56α in regulating cardiac responses to β-adrenergic stimulation *in vivo*

Sarah-Lena Puhl[1,2,*], Kate L Weeks[1,3,*], Alican Güran[1], Antonella Ranieri[1], Peter Boknik[4], Uwe Kirchhefer[4], Frank Ulrich Müller[4], Metin Avkiran[1]

[1] King's College London British Heart Foundation Centre of Research Excellence, School of Cardiovascular Medicine and Sciences, St Thomas' Hospital, Westminster Bridge Road, London SE1 7EH, United Kingdom; [2] Institute for Cardiovascular Prevention, Ludwig-Maximilians-University, Pettenkoferstrasse 9b, D-80336 Munich, Germany; [3] Baker Heart and Diabetes Institute, 75 Commercial Road, Melbourne VIC 3004, Australia; [4] Institut für Pharmakologie und Toxikologie, Universitätsklinikum Münster, Domagkstrasse 12, D-48149 Münster, Germany

* Authors contributed equally to this work.

Supplementary methods:

Isolation of adult mouse ventricular myocytes for immunoblot analyses

Ventricular myocytes from male 10-week old WT and HOM mice were isolated via collagenase digestion of Langendorff-perfused hearts, as previously described for rat hearts.[1] Myocytes in modified M199 medium (M199 (Gibco 22350-029) supplemented with 2 mM L-carnitine, 5 mM creatine, 5 mM taurine and penicillin/streptomycin) were plated on laminated 6-well cell culture plates (4 wells per mouse heart). The media was replaced 2 hours post-plating to remove dead/unattached cells, and the cells incubated at 37°C, 5% CO_2 for an additional hour. Cells were treated with 10 nM isoproterenol (DL-Isoproterenol hydrochloride, Sigma) or vehicle for 10 minutes prior to cell lysis, SDS-PAGE and immunoblotting, as described in the main text.

References:

1. Snabaitis AK, Muntendorf A, Wieland T and Avkiran M. Regulation of the extracellular signal-regulated kinase pathway in adult myocardium: differential roles of $G_{q/11}$, G_i and $G_{12/13}$ proteins in signalling by a_1-adrenergic, endothelin-1 and thrombin-sensitive protease-activated receptors. *Cellular Signalling*. 2005;17:655-64.

Supplementary Figure 1: Protein phosphorylation in isolated cardiomyocytes from adult wildtype and B56α-deficient mice following acute β-adrenergic stimulation. (A) Abundance of non-phosphorylated (0P), mono-phosphorylated (1P) and bis-phosphorylated (2P) cardiac troponin I (cTnI) in isolated cardiomyocytes from adult WT and HOM mice, as assessed by phosphate affinity (PhosTag) SDS-PAGE and immunoblotting. Cells were treated with 10 nM isoproterenol or vehicle for 10 minutes. 0P, 1P and 2P phospho-moieties are expressed as a percentage of total cTnI, which is the sum of the signals in each lane. Lines show mean ± SE (n=11 per group); *P<0.05 (two-way ANOVA with Tukey's post-hoc test). Phosphorylation of (B) cardiac myosin binding protein C (cMyBPC) at Ser282 and Ser302, (C) phospholamban (PLB) at Ser16 and (D) ryanodine receptor 2 (RyR2) at Ser2808 and Ser2814 in isolated cardiomyocytes from adult WT and HOM mice, as assessed by immunoblotting. Cells were treated with 10 nM isoproterenol or vehicle for 10 minutes. Lines show mean ± SE (n=6-10 per group); *P<0.05 (two-way ANOVA with Tukey's post-hoc test).

Supplementary Figure 1

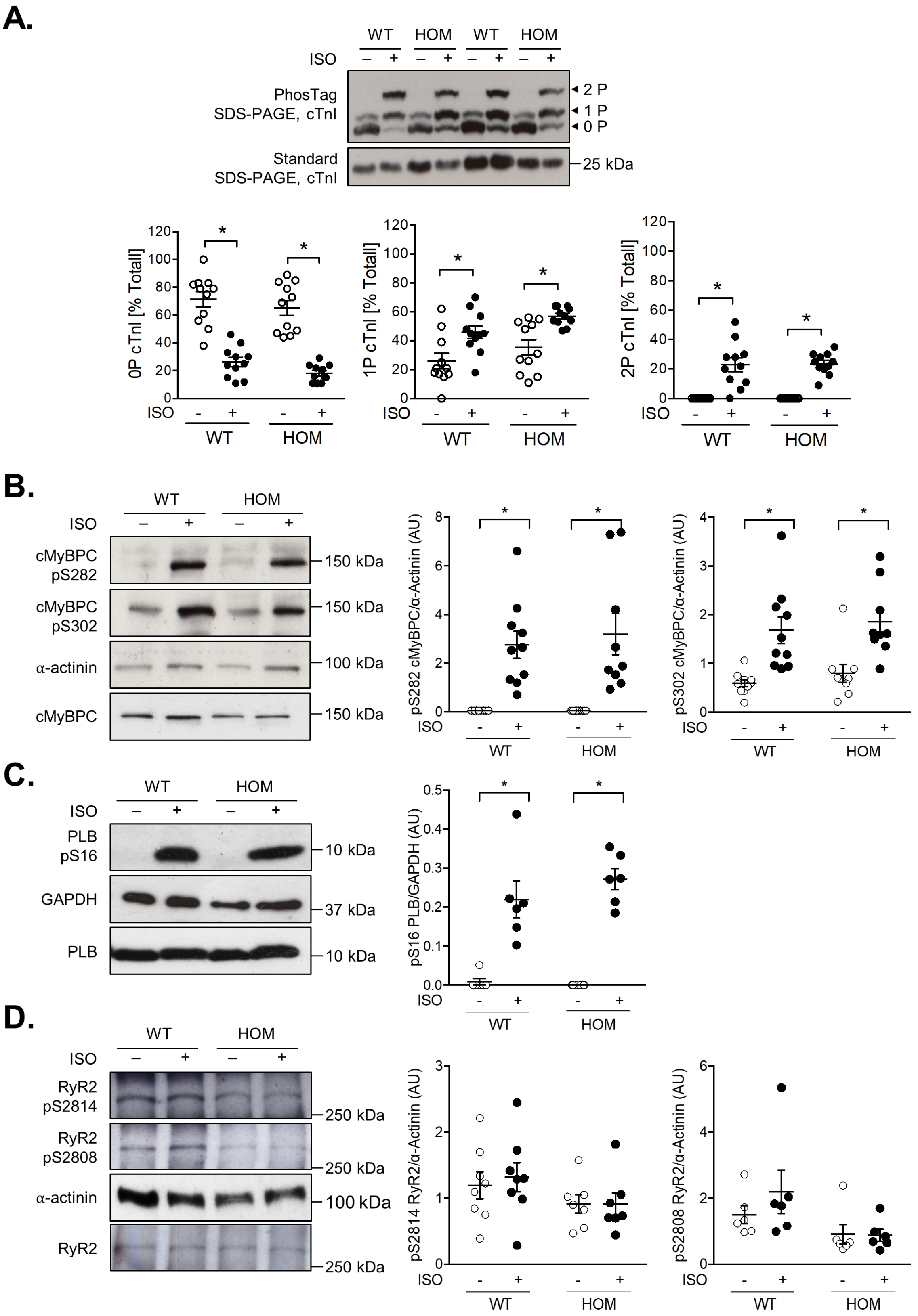

Supplementary Table 1: Basal phenotype of male mice, aged 2 months, determined via echocardiography

		WT (n=20)	HET (n=28)	HOM (n=28)
**IVS [mm]**	diastolic	0.69 ± 0.02	0.70 ± 0.01	0.67 ± 0.02
	systolic	0.95 ± 0.03	0.98 ± 0.02	1.00 ± 0.03
**LVAW [mm]**	diastolic	0.71 ± 0.02	0.70 ± 0.02	0.69 ± 0.02
	systolic	1.01 ± 0.03	1.00 ± 0.02	0.99 ± 0.02
**LVPW [mm]**	diastolic	0.78 ± 0.03	0.77 ± 0.02	0.81 ± 0.02
	systolic	1.20 ± 0.04	1.18 ± 0.03	1.07 ± 0.05
**LVID [mm]**	diastolic	3.97 ± 0.10	4.02 ± 0.07	4.14 ± 0.08
	systolic	2.74 ± 0.11	2.76 ± 0.08	2.99 ± 0.10
**LVvol [µl]**	diastolic	70.04 ± 3.88	71.78 ± 2.71	76.73 ± 3.17
	systolic	29.56 ± 2.74	30.76 ± 2.08	36.48 ± 2.83
**EF [%]**		59.1 ± 2.23	58.12 ± 1.47	54.37 ± 2.12
**FS [%]**		31.29 ± 1.50	30.49 ± 0.97	28.31 ± 1.38
**SV [µl]**		40.48 ± 1.96	41.02 ± 1.19	40.54 ± 1.13
**CO [ml min^{-1}]**		21.45 ± 1.17	21.98 ± 0.68	20.78 ± 0.72
**Heart rate [bpm]**		530 ± 12	538 ± 10	516 ± 13

Mean ± SE, no significant differences between genotypes by one-way ANOVA.

Supplementary Table 2: Basal phenotype of female mice, aged 2 months, determined via echocardiography

		WT (n=8)	HET (n=10)	HOM (n=9)
**IVS [mm]**	**diastolic**	0.65 ± 0.04	0.62 ± 0.02	0.62 ± 0.02
	**systolic**	0.95 ± 0.07	0.88 ± 0.03	0.90 ± 0.02
**LVAW [mm]**	**diastolic**	0.60 ± 0.03	0.60 ± 0.02	0.61 ± 0.02
	**systolic**	0.94 ± 0.07	0.84 ± 0.04	0.87 ± 0.04
**LVPW [mm]**	**diastolic**	0.69 ± 0.04	0.70 ± 0.03	0.69 ± 0.06
	**systolic**	1.05 ± 0.05	1.03 ± 0.05	0.98 ± 0.07
**LVID [mm]**	**diastolic**	3.81 ± 0.10	3.98 ± 0.12	3.87 ± 0.12
	**systolic**	2.62 ± 0.13	2.86 ± 0.13	2.84 ± 0.12
**LVvol [µl]**	**diastolic**	63.00 ± 3.69	70.19 ± 5.00	65.55 ± 4.63
	**systolic**	25.85 ± 2.83	32.20 ± 3.57	31.24 ± 3.26
**EF [%]**		59.57 ± 3.64	54.87 ± 2.48	52.94 ± 2.44
**FS [%]**		31.55 ± 2.51	28.27 ± 1.55	26.94 ± 1.53
**SV [µl]**		37.15 ± 2.65	37.99 ± 2.41	34.32 ± 2.19
**CO [ml min^{-1}]**		18.71 ± 1.18	17.03 ± 1.15	15.91 ± 1.26
**Heart rate [bpm]**		508 ± 16	452 ± 20	465 ± 28

Mean ± SE, no significant differences between genotypes by one-way ANOVA.

Supplementary Table 3: Basal phenotype of male mice, aged 2 months, determined via gravimetry

	WT	HET	HOM
Body dimensions			
**n**	**6**	**11**	**11**
**Body weight [g]**	21.5 ± 1.5	21.7 ± 0.7	22.6 ± 0.8
**Tibia length [mm]**	15.8 ± 0.3	15.6 ± 0.1	15.9 ± 0.1
Heart chamber weights			
**n**	**6**	**11**	**11**
**Whole heart [mg]**	108.6 ± 7.7	116.0 ± 4.2	124.1 ± 5.6
**Atria [mg]**	6.3 ± 0.5	7.2 ± 0.6	8.0 ± 0.6
**Ventricles [mg]**	102.3 ± 7.3	108.8 ± 4.0	116.1 ± 5.2
Wet organ weights			
**n**	**6**	**6**	**6**
**Lungs [mg]**	123.0 ± 4.3	129.1 ± 5.0	125.7 ± 3.4
**Liver [mg]**	1116 ± 74	1160 ± 98	1098 ± 78
**Kidney [mg]**	143.6 ± 10.4	141.5 ± 5.5	144.6 ± 4.2
**Spleen [mg]**	60.5 ± 4.1	64.3 ± 3.3	62.7 ± 3.4
Dry organ weights			
**n**	**6**	**6**	**6**
**Lungs [mg]**	27.9 ± 1.2	29.4 ± 1.3	28.1 ± 0.7
**Liver [mg]**	357 ± 12	411 ± 36	388 ± 34
**Kidney [mg]**	38.8 ± 1.9	39.4 ± 1.5	38.8 ± 1.4
**Spleen [mg]**	13.9 ± 0.8	15.4 ± 0.9	14.8 ± 0.9

Mean ± SE, no significant differences between genotypes by one-way ANOVA.

Supplementary Table 4: Basal phenotype of female mice, aged 2 months, determined via gravimetry

	WT	HET	HOM
Body dimensions			
**n**	**8**	**10**	**9**
**Body weight [g]**	18.2 ± 0.6	18.4 ± 0.5	16.9 ± 0.5
**Tibia length [mm]**	15.4 ± 0.2	15.2 ± 0.1	15.2 ± 0.1
Heart chamber weights			
**n**	**8**	**10**	**9**
**Whole heart [mg]**	96.3 ± 5.6	99.0 ± 4.8	91.1 ± 2.8
**Atria [mg]**	6.0 ± 0.3	6.2 ± 0.7	6.2 ± 0.4
**Ventricles [mg]**	90.3 ± 5.4	92.8 ± 4.3	84.9 ± 2.6
Wet organ weights			
**n**	**8**	**6-7**	**4-8**
**Lungs [mg]**	126.3 ± 4.7	124.7 ± 2.7	118.9 ± 4.0
**Liver [mg]**	811 ± 48	948 ± 65	981 ± 93
**Kidney [mg]**	109.6 ± 5.2	111.5 ± 4.2	110.2 ± 5.9
**Spleen [mg]**	72.3 ± 4.6	86.0 ± 6.2	71.9 ± 5.5
Dry organ weights			
**n**	**8**	**6**	**4**
**Lungs [mg]**	28.5 ± 1.0	28.2 ± 0.7	28.1 ± 1.0
**Liver [mg]**	259 ± 15	322 ± 20	345 ± 35*
**Kidney [mg]**	28.6 ± 1.0	29.2 ± 1.1	29.3 ± 1.6
**Spleen [mg]**	16.7 ± 1.1	19.5 ± 1.3	16.9 ± 1.0

Mean ± SE, *p<0.05 vs. WT, one-way ANOVA with Tukey's post-hoc test.

Supplementary Table 5: Basal phenotype of male mice, aged 6 months, determined via gravimetry

	WT	HET	HOM
Body dimensions			
**n**	**8**	**8**	**11**
**Body weight [g]**	34.9 ± 1.5	34.2 ± 1.3	33.2 ± 1.0
**Tibia length [mm]**	16.7 ± 0.1	16.7 ± 0.1	16.9 ± 0.1
Heart chamber weights [mg]			
**n**	**8**	**8**	**11**
**Whole heart**	173.8 ± 4.6	165.8 ± 4.8	155.0 ± 6.5
**Atria**	9.3 ± 0.7	9.4 ± 0.4	7.9 ± 0.3
**Ventricles**	164.5 ± 4.2	156.5 ± 4.5	147.1 ± 6.3
Wet organ weights [mg]			
**n**	**8**	**8**	**11**
**Lungs**	164.3 ± 3.9	161.3 ± 3.0	146.6 ± 2.9*†
**Liver**	1341 ± 127	1517 ± 57	1659 ± 81
**Kidney**	223.3 ± 7.2	224.1 ± 5.7	211.4 ± 7.5
**Spleen**	94.0 ± 4.0	100.4 ± 11.7	80.3 ± 6.8
Wet organ weights normalized to tibia length [mg/mm]			
**n**	**8**	**8**	**11**
**Whole heart**	10.43 ± 0.29	9.95 ± 0.30	9.19 ± 0.38*
**Atria**	0.56 ± 0.04	0.56 ± 0.02	0.47 ± 0.02
**Ventricles**	9.87 ± 0.26	9.39 ± 0.28	8.72 ± 0.36
**Lungs**	9.85 ± 0.24	9.67 ± 0.18	8.69 ± 0.18*†
**Liver**	80.18 ± 7.27	91.00 ± 3.41	93.39 ± 4.76
**Kidney**	13.40 ± 0.47	13.45 ± 0.36	12.53 ± 0.43
**Spleen**	5.64 ± 0.25	6.03 ± 0.72	4.76 ± 0.40

Mean ± SE, *p<0.05 vs. WT, †p<0.05 vs. HET, one-way ANOVA with Tukey's post-hoc test.

Supplementary Table 6: Basal phenotype of male mice, aged 6 months, determined via echocardiography

		WT (n=6)	HET (n=8)	HOM (n=9)
**IVS [mm]**	**diastolic**	0.79 ± 0.04	0.72 ± 0.03	0.66 ± 0.02*
	**systolic**	1.14 ± 0.07	1.01 ± 0.04	0.95 ± 0.04*
**LVAW [mm]**	**diastolic**	0.77 ± 0.03	0.75 ± 0.03	0.73 ± 0.04
	**systolic**	1.08 ± 0.06	1.06 ± 0.02	0.93 ± 0.06
**LVPW [mm]**	**diastolic**	0.81 ± 0.04	0.86 ± 0.03	0.83 ± 0.05
	**systolic**	1.11 ± 0.08	1.17 ± 0.05	1.09 ± 0.08
**LVID [mm]**	**diastolic**	4.84 ± 0.19	4.65 ± 0.08	4.73 ± 0.08
	**systolic**	3.73 ± 0.21	3.59 ± 0.09	3.66 ± 0.11
**LVvol [µl]**	**diastolic**	111.10 ± 10.12	99.82 ± 3.72	104.50 ± 4.18
	**systolic**	60.05 ± 7.40	54.56 ± 3.22	57.10 ± 4.49
**EF [%]**		46.63 ± 3.69	45.48 ± 2.12	45.74 ± 2.41
**FS [%]**		23.55 ± 2.24	22.69 ± 1.29	22.90 ± 1.37
**SV [µl]**		51.00 ± 4.60	45.26 ± 2.12	47.37 ± 2.38
**CO [ml min^{-1}]**		26.86 ± 1.56	21.76 ± 1.34	22.07 ± 1.74
**Heart rate [bpm]**		507 ± 32	479 ± 14	477 ± 24

Mean ± SE; *p<0.05 vs. WT, one-way ANOVA with Tukey's post-hoc test.

Supplementary Table 7: Basal phenotype of female mice, aged 6 months, determined via gravimetry

	WT	HET	HOM
Body dimensions			
**n**	**7**	**8**	**8**
**Body weight [g]**	25.8 ± 1.7	28.6 ± 1.8	27.1 ± 1.4
**Tibia length [mm]**	16.9 ± 0.1	16.9 ± 0.1	17.2 ± 0.1
Heart chamber weights [mg]			
**n**	**7**	**8**	**8**
**Whole heart**	127.6 ± 6.7	120.6 ± 4.1	135.3 ± 5.5
**Atria**	6.2 ± 0.4	5.8 ± 0.3	8.5 ± 0.8*†
**Ventricles**	121.4 ± 6.4	114.8 ± 4.1	126.8 ± 5.4
Wet organ weights [mg]			
**n**	**7**	**8**	**8**
**Lungs**	148.4 ± 3.7	150.4 ± 3.6	158.2 ± 5.2
**Liver**	1151 ± 107	1426 ± 85	1242 ± 115
**Kidney**	157.9 ± 8.9	144.5 ± 5.6	169.8 ± 14.1
**Spleen**	103.1 ± 9.2	88.6 ± 7.2	97.3 ± 6.4
Wet organ weights normalized to tibia length [mg/mm]			
**n**	**7**	**8**	**8**
**Whole heart**	7.57 ± 0.38	7.15 ± 0.21	7.88 ± 0.30
**Atria**	0.37 ± 0.02	0.35 ± 0.02	0.50 ± 0.05*†
**Ventricles**	7.20 ± 0.36	6.80 ± 0.22	7.39 ± 0.29
**Lungs**	8.80 ± 0.17	8.91 ± 0.17	9.22 ± 0.26
**Liver**	68.15 ± 5.91	84.59 ± 5.02	72.23 ± 6.38
**Kidney**	9.37 ± 0.53	8.56 ± 0.32	9.88 ± 0.78
**Spleen**	5.31 ± 0.78	5.26 ± 0.43	5.66 ± 0.34

Mean ± SE, *p<0.05 vs. WT, †p<0.05 vs. HET, one-way ANOVA with Tukey's post-hoc test.

Supplementary Table 8: Basal phenotype of female mice, aged 6 months, determined via echocardiography

		WT (n=8)	HET (n=8)	HOM (n=8)
**IVS [mm]**	**diastolic**	0.68 ± 0.03	0.65 ± 0.03	0.66 ± 0.01
	**systolic**	1.03 ± 0.04	0.92 ± 0.04	0.93 ± 0.02
**LVAW [mm]**	**diastolic**	0.65 ± 0.04	0.63 ± 0.03	0.66 ± 0.04
	**systolic**	0.92 ± 0.06	0.89 ± 0.05	0.95 ± 0.05
**LVPW [mm]**	**diastolic**	0.87 ± 0.06	0.84 ± 0.03	0.73 ± 0.04
	**systolic**	1.18 ± 0.07	1.21 ± 0.06	1.03 ± 0.05
**LVID [mm]**	**diastolic**	4.13 ± 0.10	4.10 ± 0.08	4.49 ± 0.14
	**systolic**	3.05 ± 0.12	3.04 ± 0.09	3.39 ± 0.12
**LVvol [µl]**	**diastolic**	76.11 ± 4.33	74.58 ± 3.15	92.85 ± 6.73
	**systolic**	37.28 ± 3.66	36.48 ± 2.73	47.85 ± 4.38
**EF [%]**		51.09 ± 3.41	51.32 ± 2.21	48.72 ± 1.63
**FS [%]**		26.04 ± 2.14	26.01 ± 1.38	24.52 ± 0.98
**SV [µl]**		38.83 ± 3.12	38.09 ± 1.72	44.99 ± 2.99
**CO [ml min^{-1}]**		16.53 ± 1.87	20.60 ± 0.83	23.81 ± 2.45
**Heart rate [bpm]**		462 ± 25	512 ± 12	515 ± 22

Mean ± SE; *p<0.05 vs. WT, one-way ANOVA with Tukey’s post-hoc test.

Supplementary Table 9: Effect of acute β-AR stimulation on cardiac phenotype in male mice, aged 2 months, determined via echocardiography

		WT (n=8-10)		HET (n=10)		HOM (n=9-10)	
	**DOB**	**-**	**+**	**-**	**+**	**-**	**+**
**IVS [mm]**	**diastolic**	0.73 ± 0.03	0.81 ± 0.03	0.71 ± 0.02	0.82 ± 0.03	0.68 ± 0.03	0.77 ± 0.03
	**systolic**	0.98 ± 0.05	1.21 ± 0.04*	0.99 ± 0.04	1.19 ± 0.04*	0.92 ± 0.02	1.21 ± 0.03*
**LVAW [mm]**	**diastolic**	0.75 ± 0.02	0.83 ± 0.02	0.71 ± 0.02	0.82 ± 0.03	0.73 ± 0.04	0.76 ± 0.04
**LVPW [mm]**	**diastolic**	0.82 ± 0.03	0.97 ± 0.04	0.85 ± 0.03	0.93 ± 0.03	0.78 ± 0.02	0.86 ± 0.03
**LVID [mm]**	**diastolic**	3.89 ± 0.10	3.49 ± 0.12	3.97 ± 0.09	3.41 ± 0.09*	4.04 ± 0.12	3.86 ± 0.09
**LVvol [µl]**	**diastolic**	66.02 ± 3.95	51.55 ± 4.33	69.19 ± 3.69	48.50 ± 3.14*	72.46 ± 4.90	64.86 ± 3.40
	**systolic**	27.52 ± 2.42	14.63 ± 2.35*	29.67 ± 2.58	12.05 ± 1.46*	32.45 ± 4.05	22.22 ± 2.52
**SV [µl]**		38.50 ± 1.76	36.92 ± 2.48	39.52 ± 1.75	36.45 ± 2.20	40.00 ± 1.42	42.65 ± 1.53
**CO [ml min^{-1}]**		19.67 ± 2.36	23.27 ± 1.63	22.68 ± 0.86	23.07 ± 1.42	22.01 ± 1.02	26.57 ± 0.93

Mean ± SE; *$p<0.05$ vs. Vehicle (- DOB), †$p<0.05$ vs. HET, two-way ANOVA with Tukey's post-hoc test.

Supplementary Table 10: Effect of sustained β-AR stimulation on cardiac phenotype in male mice, aged 2 months, determined via echocardiography

		WT (n=7-8)		HOM (n=6-7)	
	ISO	-	+	-	+
IVS [mm]	diastolic	0.71 ± 0.02	0.84 ± 0.05	0.67 ± 0.04	0.82 ± 0.02
	systolic	0.95 ± 0.05	1.09 ± 0.06	0.92 ± 0.05	1.08 ± 0.05
LVvol [µl]	diastolic	95.64 ± 7.01	97.40 ± 9.99	91.22 ± 6.00	87.49 ± 9.14
	systolic	47.11 ± 5.64	51.16 ± 7.38	47.77 ± 6.87	42.49 ± 7.45
SV [µl]		48.52 ± 2.66	46.24 ± 4.02	43.45 ± 2.35	45.00 ± 2.21
CO [ml min^{-1}]		24.88 ± 2.15	27.14 ± 2.29	19.76 ± 1.87	26.94 ± 1.56

Mean ± SE; *p<0.05 vs. Vehicle (- ISO), two-way ANOVA with Tukey's post-hoc test

Supplementary Table 11: Effect of sustained β-AR stimulation on cardiac phenotype in male mice, aged 2 months, determined via gravimetry

	WT		HOM	
**ISO**	**-**	**+**	**-**	**+**
**n**	**7**	**8**	**5**	**10**
Body dimensions				
**Body weight [g]**	24.9 ± 0.7	28.5 ± 0.8*	25.0 ± 1.4	26.6 ± 0.8
**Tibia length [mm]**	16.1 ± 0.1	16.1 ± 0.1	16.1 ± 0.1	16.0 ± 0.1
Heart chamber weights [mg]				
**Heart**	128.0 ± 3.1	169.1 ± 8.4*	133.8 ± 9.7	162.8 ± 7.7
**Atria**	7.6 ± 0.5	10.7 ± 1.0	8.0 ± 0.6	11.1 ± 1.2
Wet organ weights [mg]				
**Lungs**	145.0 ± 3.6	152.0 ± 6.9	131.6 ± 6.7	162.3 ± 5.7*
**Liver**	1242 ± 128	1228 ± 83	1278 ± 53	1349 ± 64
**Kidney**	169.6 ± 6.9	179.0 ± 11.6	168.5 ± 10.5	170.6 ± 9.4
**Spleen**	94.3 ± 8.6	92.7 ± 10.3	74.7 ± 7.4	89.5 ± 13.5
Wet organ weights normalized to tibia length [mg/mm]				
**Heart**	7.98 ± 0.21	10.50 ± 0.47*	8.29 ± 0.57	10.18 ± 0.47*
**Atria**	0.48 ± 0.03	0.67 ± 0.06	0.49 ± 0.04	0.70 ± 0.08
**Lungs**	9.04 ± 0.26	9.45 ± 0.43	8.16 ± 0.40	10.15 ± 0.33*
**Liver**	77.23 ± 7.67	76.23 ± 4.82	79.18 ± 3.30	84.37 ± 3.85
**Kidney**	10.57 ± 0.42	11.11 ± 0.66	10.44 ± 0.61	10.66 ± 0.55
**Spleen**	5.90 ± 0.58	5.75 ± 0.61	4.63 ± 0.46	5.60 ± 0.86
Fluid content of organs [mg]				
**Lungs**	110.1 ± 2.1	116.5 ± 5.3	101.0 ± 6.1	126.3 ± 4.6*
**Liver**	844 ± 90	825 ± 58	886 ± 52	940 ± 45
**Kidney**	125.7 ± 4.9	132.2 ± 8.3	125.5 ± 6.1	125.5 ± 8.7
**Spleen**	71.8 ± 6.5	69.9 ± 7.7	56.7 ± 6.0	71.3 ± 12.7

Mean ± SE; *p<0.05 vs. Vehicle (- ISO), two-way ANOVA with Tukey’s multiple comparison test.
